# Contrast Circulation Time to Assess Right Ventricular Dysfunction in Pulmonary Embolism: A Retrospective Pilot Study

**DOI:** 10.1371/journal.pone.0159674

**Published:** 2016-08-23

**Authors:** Gregor John, Alexandra Platon, Pierre-Alexandre Poletti, Arnaud Perrier, Karim Bendjelid

**Affiliations:** 1 Department of Internal medicine, Hôpital neuchâtelois, Chasseral 20, 2300 La Chaux-de-Fonds, Switzerland; 2 Department of Internal Medicine, Rehabilitation and Geriatrics, Geneva University Hospitals (HUG), Gabrielle-Perret-Gentil 4, 1205 Geneva, Switzerland; 3 Department of radiology, Emergency-room radiology unit, Geneva University Hospitals (HUG), Gabrielle-Perret-Gentil 4, 1205 Geneva, Switzerland; 4 Geneva Faculty of Medicine, Michel-Servet 1, 1206 Geneva, Switzerland; 5 Intensive Care Service, Geneva University Hospitals, and Geneva Faculty of Medicine, Gabrielle Perret-Gentil 4, 1211 Geneva, Switzerland; 6 Geneva Hemodynamic Research Group, Geneva Faculty of Medicine, Michel-Servet 1, 1206 Geneva, Switzerland; Universite de Bretagne Occidentale, FRANCE

## Abstract

**Objective:**

To optimize enhancement of pulmonary arteries and facilitate diagnosis of pulmonary embolism (PE), modern computed tomography angiography (CTA) contains a contrast bolus tracking system. We explored the diagnostic accuracy of the time-intensity curves given by this automated system to identify right ventricular dysfunction (RVD) in acute PE.

**Methods:**

114 CTAs with a diagnosis of PE were reviewed. RVD was defined as right-to-left ventricular diameter ratio of 1 or greater. Four parameters on time-intensity curves were identified. Parameters between CTAs with and those without RVD were compared with the Wilcoxon rank-sum test. The ability of the four parameters to discriminate patients with RVD was explored by compiling the area under the operating curves (AUC).

**Results:**

The time needed by the contrast media to reach the pulmonary artery [8 seconds (IQR: 7–9) versus 7 seconds (IQR: 6–8), p<0.01], the time needed to reach 40 Hounsfield units (HU) [11 seconds (IQR: 8.5–14) versus 9.5 seconds (IQR: 8–10.5), p<0.01], and the contrast intensity reached after 10 seconds [19 HU (IQR: 4–67) versus 53 HU (IQR: 32–80), p<0.05] were all statistically different between CTA with and CTA without RVD. Those three parameters changed gradually across severity categories of RVD (p<0.05 for trend). Their AUC to identify RVD ranged from 0.63 to 0.66. The slope of contrast intensity over time was not informative: [31 HU/s (IQR: 20–57) in CTA with, compared to 36 HU/s (IQR: 22.5–53) in CTA without RVD, p = 0.60].

**Conclusion:**

Several parameters of the time-intensity curve obtained by the bolus tracking system are associated with RVD assessed on CTA images. Of those, the time needed to reach a predefined threshold seems to be the easiest to obtain in any CTA without additional processing time or contrast injection. However, the performance of those parameters is globally low.

## Introduction

Risk stratification is a cornerstone in the care of pulmonary embolism (PE) [1–4]. It guides treatment intensity (thrombolysis and anticoagulants versus anticoagulants alone [5]), and allows for the selection of patients who need close hemodynamic monitoring in the ICU, those who can be admitted in a general ward and those who can be safely treated as outpatients [6].

Normotensive patients with right ventricular dysfunction (RVD) have twice the 30-day mortality rate than patients without RVD [7–9]. The present heart dysfunction may be assessed by different imaging techniques and/or biomarkers [2, 10]. However, despite its usefulness in risk stratification, any strategy to identify RVD has some relative drawbacks: echocardiography requires skilled specialists and is not available around the clock in many hospitals [11], some CT signs are time consuming or could be dependent on manipulations of workstation by the radiologist [12, 13], and biomarkers add extra cost that limit their use.

Computed tomography angiography (CTA) of the chest is currently the preferred method to diagnose PE. Moreover, CTA signs such as the right-to-left ventricular ratio, or reflux of contrast media in the hepatic veins are now well recognized and can help in patient management. Increased right-to-left ventricular ratio correlates with RVD assessed on echocardiography [14–16], all-causes mortality, PE-related mortality, and other composite outcomes [13, 17, 18]. In RDV diagnosed by echocardiography, the sensitivity and specificity of right-to-left ventricular ratio (≥1) measured by CTA are 91% (95% CI 72–99%) and 79% (95% CI 69%-87%), respectively [19]. Furthermore, CTA right-to-left ventricular ratio performs as well as echocardiography ratio to predict death after PE [2, 13, 17, 18, 20] and was used interchangeably as inclusion criteria of intermediate-high risk patients in the large PEITHO study [21]. Thus, the American Heart Association, and the European Society of Cardiology guidelines, both recommend using either echocardiography or CTA for risk stratification in PE (a class IIa recommendation with level B evidence) [2, 4].

To synchronize image acquisition with the adequate filling of the pulmonary artery by contrast medium, modern CTA are equipped with an automatic contrast tracking system. This system captures the intensity of contrast in the pulmonary trunk (measured in Hounsfield units) over time and allows for determining the starting point of data acquisition when the media enhances the pulmonary artery. The spread of the contrast media from the periphery veins to the pulmonary arteries is a dynamic process, which reflects mainly the right heart blood flow. Therefore, the time needed by contrast media to reach the pulmonary trunk should be longer, and the slope of increasing contrast intensity over time should be flatter, in the event of increased right heart afterload and/or depressed right ventricular function [22, 23]. This approach could add to the prognostic information provided by diagnostic CTA in case of pulmonary embolism. A recently published retrospective study suggested that characteristics of contrast enhancement over time could correlate with death [24]. Thus we sought to explore the ability of immediately available parameters obtained by the tracking system to identify RVD related to acute pulmonary embolism.

## Subjects/Patients and Methods

We conducted a retrospective study on all consecutive CTAs of the chest done in the work-up of pulmonary embolism in the emergency room of a single tertiary hospital in Switzerland from the 16^th^ of September 2012 to the 31^th^ of December 2013. All analyses were performed on anonymized CT angiograms, unrelated to participants’ medical charts. The Ethics committee approved the study. The informed consent was waived due to the retrospective design and limited patient information.

### Participants and CTA

**Fig 1:**
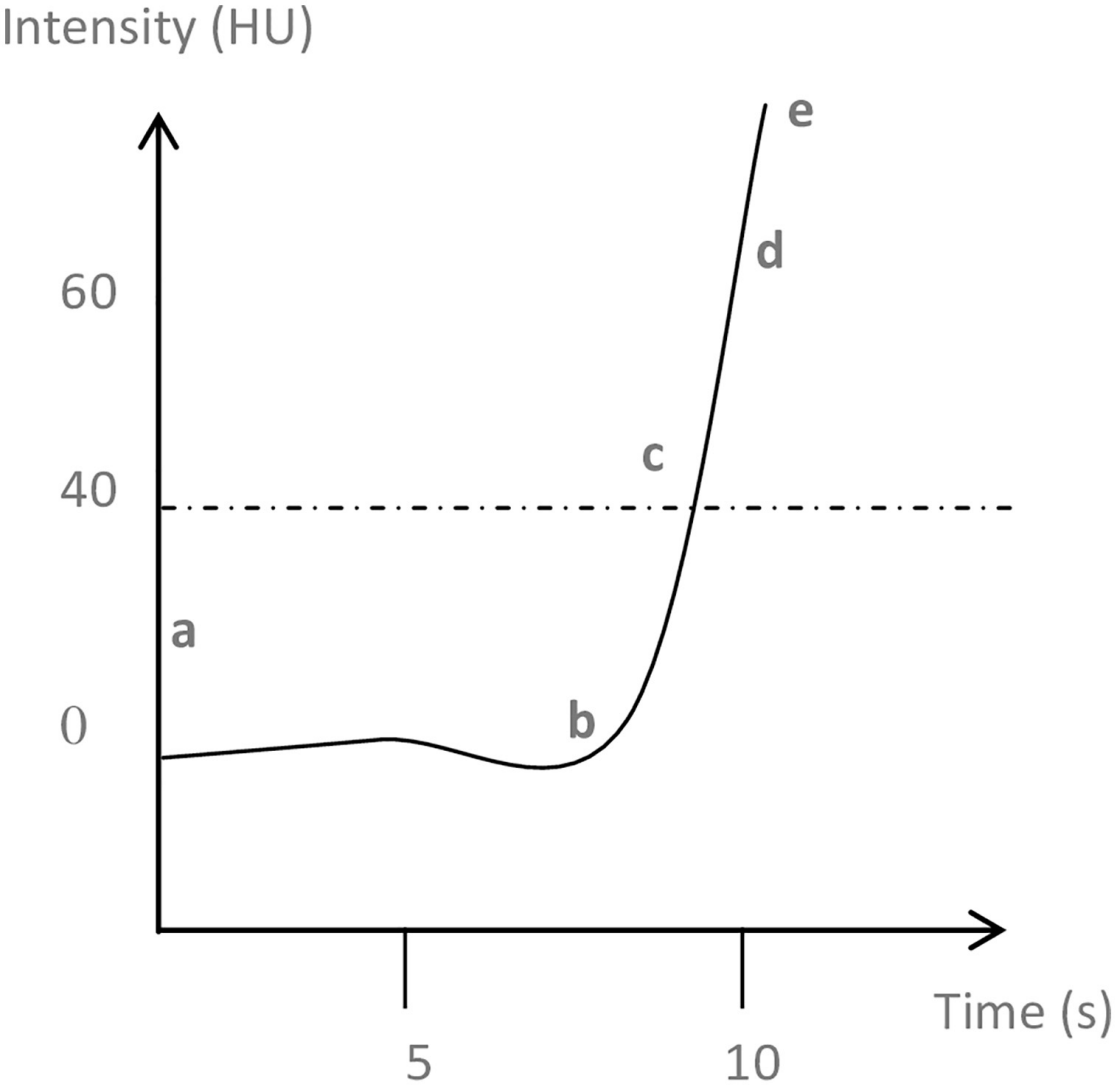
Schematic time-intensity curve given by the bolus tracking system. From the injection of contrast into the peripheral vein (point a), until the contrast media reaches the pulmonary artery (b), the curve is flat. After the inflection point (b), there is a brisk rise in intensity measured by the system, and the intensity reaches a predefined threshold to start the sequence of data acquisition (40UH, point (c)). The intensity slope is calculated by subtracting the intensity measured at the inflection point (b) to the highest intensity measured (e) divided by the time between those two points. The point (d) is the intensity reached at 10 seconds. ROI: region of interest; HU: Hounsfield units; s: seconds.

All CTA scans were performed on a 64-rows GE 750 HD CT (Discovery 750 HD CT, GE Healthcare, Milwaukee, USA). The acquisition was made from the pulmonary apex to the diaphragm, after an intravenous injection of 60 ml of contrast media (Iohexol, 350 mg I/ml, GE Healthcare AG), at a 3.5 ml/sec rate, using the following parameters: 100 kV tube voltage, variable tube charge 150–450 mAs, 1.25–0.8 slice collimation, pitch: 0.9, rotation time: 0.4 sec. The contrast medium injection was performed using the bolus tracking system (“SmartPrep” protocol, GE Healthcare): after starting the intravenous contrast-injection, repeated scans were made at the level of the main pulmonary artery, at a low tube current (40 mA), with a monitoring delay of 4 seconds, and an Interscan Delay (ISD) of 1 to 2 seconds. A circular region of interest (ROI) was placed on the main pulmonary artery and the CT technician manually initiated the acquisition when the pre-set threshold level of enhancement of 40 Hounsfield Unit (HU) was reached in the ROI. This measure of enhancement over time was charted on a graph (time-intensity curve; Fig 1), and could be uploaded with the CTA images.

The study cases were obtained by computer search in the radiology database, using the keywords “CT angiography” and “pulmonary embolism”. All patients who had a CTA done during the predefined period, a final diagnosis of pulmonary embolism, and an uploaded time-intensity curve along with the CTA images were included. Patients with a time-intensity curve that did not reach the intensity of 40HU, or that were not interpretable due to artefacts, were excluded. The patients were then divided into two groups depending on the presence or absence of RVD assessed by right-to-left ventricular ratios on CTA.

### Measurements

#### Right ventricular dysfunction

RVD was considered present if right-to-left ventricular minor axis diameter ratio was 1 or greater [2, 18]. Those axes were measured at the widest points between the inner surface of the free wall and the surface of the interventricular septum in the same images used for diagnosis, without reconstructions. The right and left ventricle diameters were therefore often measured in different CTA slices. This simple measure has been shown to be equivalent to more complex measures of the right-to-left ratio [13]. An experienced radiologist (in charge of the emergency room) and a specialist in internal medicine (GJ), with no specific expertise in radiology, each independently measured and calculated the right-to-left ratio, and the mean of both results was the final ratio.

#### Time-intensity curve

We explored four parameters on all time-intensity curves (Fig 1). Assessment of those parameters was done separately from the measures of RVD. Thus the assessors were unaware of the RVD status. The time to inflection point was the time needed by the contrast media to reach the pulmonary trunk after its injection into the peripheral vein. The time to peak was that required to reach a determined intensity (40HU) starting from the time of injection [23]. The contrast intensity slope was calculated by subtracting the intensity measured at the inflection point to the highest intensity measured divided by the length of time between those two points. The contrast intensity at 10 seconds was the intensity reached at 10 seconds or the highest intensity reached before, when the elapsed time was shorter than 10 seconds.

### Statistical analysis

A preliminary analysis of the first 20 CTA and their time-intensity curves was used to calculate the final sample size. We calculated that 108 CTA (50% with an RVD) would be necessary to detect a median difference (two tails) in the time needed to reach 40HU of 1.5 seconds between CTA with and those without RVD, with a statistical power of 90% and an alpha error of 5%.

The right-to-left ratios and all measures of the time-intensity curves were performed twice. Inter-rater agreement was assessed by a kappa statistic for dichotomous and Spearman rank correlation for continuous variables.

Comparisons of characteristics between CTAs with and without a RVD were performed using the chi-squared test or the Fisher exact test when appropriate for categorical variables. Due to the small sample size and the non-normal distribution of the continuous variables we used the Wilcoxon rank-sum test to compare continuous variables.

We divided the right-to-left ratios in three categories (<1; 1–1.4, and >1.4). The rationale for this subdivision comes from the observation that among positive right-to-left ratios (≥1), a higher ratio (>1.3 to 1.5) is encountered among unstable or more severe PE [25–27]. The trend towards RVD severity was determined through logistic regression for categorical variables (the binary variable being the dependant variable, the grade of RVD being the independent variable), and through an extension of the Wilcoxon rank-sum test for trend across ordered groups [28].

Logistic regression was used to assess the association between RVD and all four measures on time-intensity curves, and age, gender, and the presence of central pulmonary embolism. We built a parsimonious multivariate model with the stepwise backward method: we introduced all predictors associated in univariate analysis (p<0.2) in a logistic regression and removed one by one any variables not associated with RVD (and with no confounding effect), starting with the one with the greatest *p* value. The four parameters of the time-intensity curves (time to inflection point, time to peak, slope of intensity, and intensity at 10 seconds) were collinear and were introduced in separate models. Finally, only age was kept in the four adjusted models. The area under the operating curves of the four models was compared. We assessed the optimal cut point of the four time-curve continuous parameters with the Youden index (sensitivity+specificity-1). We computed sensitivity, specificity, predictive values, and likelihood ratios for each cut-off-point.

Two post hoc sensitivity analyses were performed to test the consistency of RVD assessment. First, RVD was restricted to right-to-left ratio≥1 measured by the experimented radiologist. Second, RVD was defined by a combination of right-to-left ratio≥1 and/or the presence of contrast media in the hepatic veins. Reflux of contrast media into the inferior vena cava is an indirect CTA sign of increased right ventricular pressure that can be seen in various underlying conditions [29]. Only reflux grades 4–6 (contrast media observed down to the hepatic veins) have prognostic significance and good inter-observer reproducibility [10, 30]. Thus, we only considered RVD when CTA showed contrast media in the hepatic veins. Since those results are concordant with the main analysis, they are only shown in the supplementary files (S1 and S2 Tables).

*p* values of less than 0.05 were regarded as statistically significant. All the analyses were performed using STATA statistical software, version 12.0 (StataCorp LP, Texas, USA).

## Results

**Fig 2:**
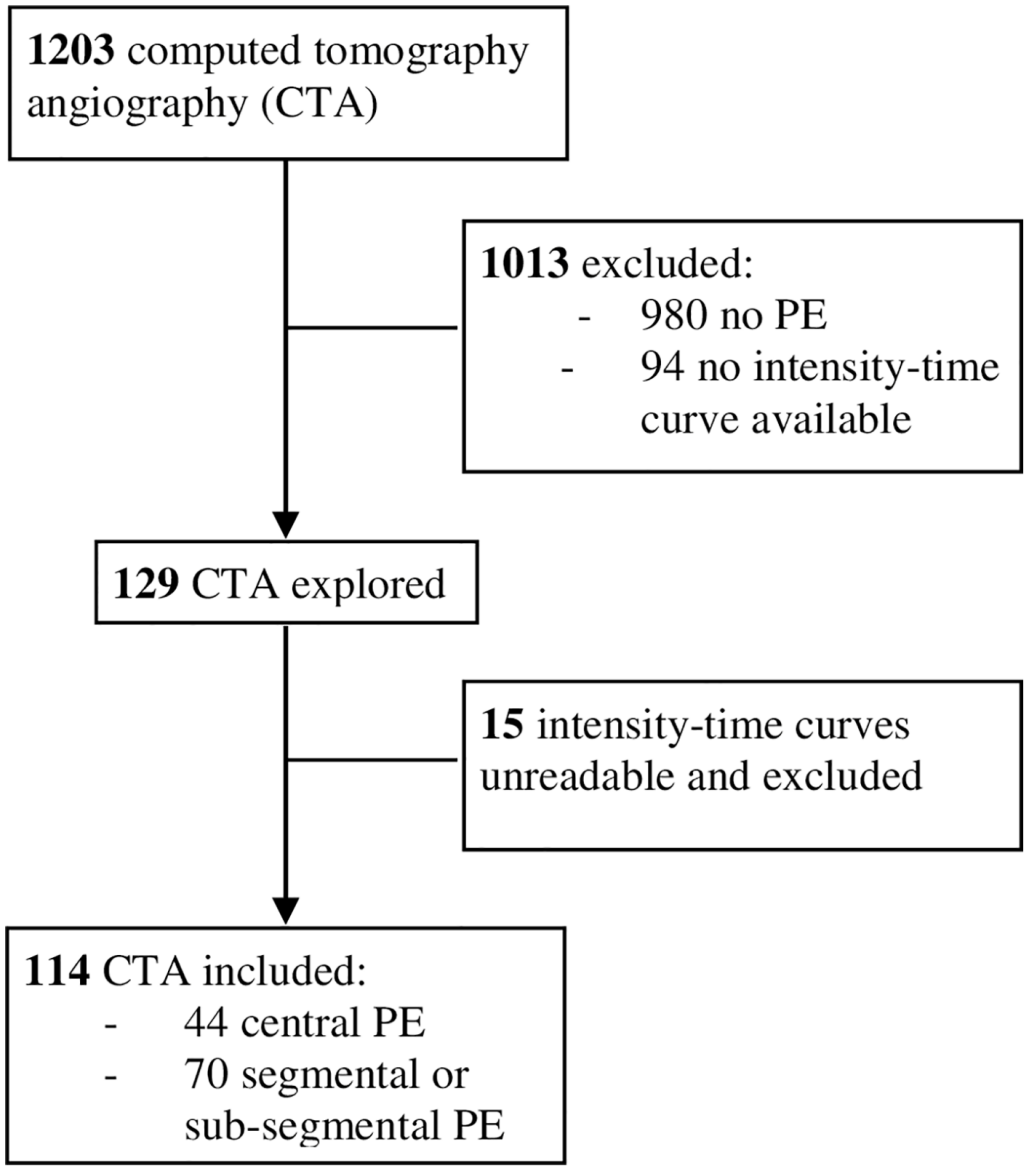
Flowchart of identified and included computed tomography angiographies (CTA).

1203 CTAs were identified between the 16^th^ of September 2012 and the 31^th^ of December 2013, and 114 had both proven pulmonary embolism and a contrast-time curve saved along with the CTA images in the computer system (Fig 2). Characteristics of the CTAs and time-intensity curves are given in Table 1. All measures except the slope of intensity were statistically different between CTAs with and without RVD (Table 1). Inter-rater agreement was excellent, except for the right-to-left diameter ratio, which was good (Table 2).

**Table 1:** Characteristics of computed tomography angiography by presence or absence of right ventricular dysfunction (RVD) defined as right-to-left ventricular ratio ≥ 1.

	RVD (N 61)	No RVD (N 53)	*P* value
Mean age (95%CI), y	73 (63–81)	61 (45–79)	0.01
Female (%)	24 (39%)	28 (53%)	0.20
Central PE (%)	30 (49%)	14 (26%)	0.02
Segmental/ sub segmental PE	31 (51%)	39 (74%)	0.02
Median (IQR) time to inflection*, s	8 (7–9)	7 (6–8)	<0.01
Median (IQR) time to 40UH^‡^, s	11 (8.5–14)	9.5 (8–10.5)	<0.01
Median (IQR) intensity at 10 seconds^†^, UH	19 (4–67)	53 (32–80)	0.01
Median (IQR) slope of intensity/time^§^, HU/s	31 (20–57)	36 (22.5–53)	0.60

* Time needed by the contrast media to reach the pulmonary trunk after its injection into the peripheral vein.

^†^ Time needed to reach a determined intensity (40HU) starting from the injection time.

^‡^ The intensity reached at 10 seconds or the highest intensity reached before, when the elapsed time was less than 10 seconds.

^§^ The intensity slope was calculated by subtracting the intensity measured at the inflection point to the highest intensity measured divided by the time between those two points.

CTA: computed tomography angiography; CI: confidence interval; HU: Hounsfield unit; IQR: interquartile range 25%-75%; PE: pulmonary embolism; RVD: right ventricular dysfunction; s: seconds; y: years.

**Table 2:** Inter-reader agreement for the right ventricular dysfunction assessment and the four time-intensity curves measures.

	Kappa	Rho	*P* value
Right-to-left ratio>1	0.64	-	<0.001
Reflux in hepatic vein	1	-	<0.001
Time to inflection point*	-	0.87	<0.001
Time to 40HU^†^	-	0.99	<0.001
Intensity at 10 seconds^‡^	-	0.66	<0.001
Slope of intensity/time^§^	-	0.98	<0.001

* Time needed by the contrast media to reach the pulmonary trunk after its injection into the peripheral vein.

^†^ Time needed to reach a determined intensity (40HU) starting from the injection time.

^‡^ The intensity reached at 10 seconds or the highest intensity reached before, when the elapsed time was less than 10 seconds.

^§^ The slope of intensity was calculated by subtracting the intensity measured at the inflection point to the highest intensity measured divided by the time between those two points.

HU: Hounsfield unit.

**Fig 3:**
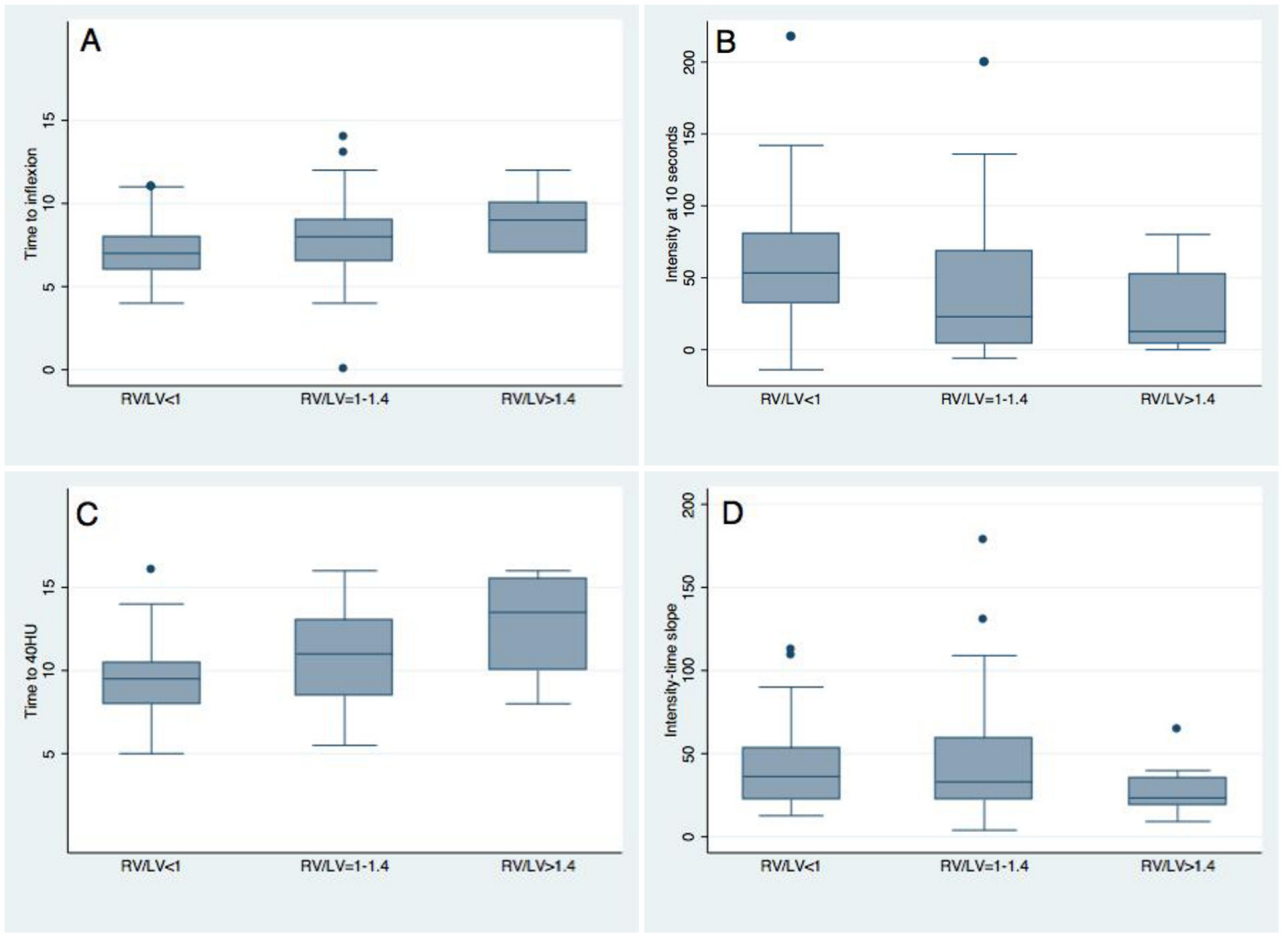
Time-intensity curve characteristics by right-to-left ventricular ratio categories. **Panel A:** time needed by the contrast media to reach the pulmonary artery (*p* value for trends <0.01). **Panel B:** intensity measured at 10 seconds after the injection of contrast media into the peripheral veins (*p* value for trends 0.02). **Panel C:** time needed by the time-intensity curve to reach 40 Hounsfield units after the injection of contrast media into the peripheral veins (*p* value for trends <0.01). **Panel D:** slope of the increasing intensity over time after the contrast media has reached the pulmonary artery (*p* value for trends 0.2). RV/LV: right-to-left ventricular ratio.

The time needed to reach the inflection point and to reach 40HU increased across the severity categories of RVD (Fig 3). The intensity reached 10 seconds after the injection of the contrast media into the peripheral vein, and the slope of increasing intensity over time decreased progressively over the severity categories of RVD (Fig 3). The latter association was not statistically significant.

In univariate logistic regression, the time to inflection point (OR 1.24; 95% CI: 1.04–1.48; *p* = 0.02), the time needed to reach 40HU (OR 1.23; 95% CI: 1.06–1.43; P<0.01), and the intensity at 10 seconds (OR 1.01; 95% CI: 1.01–1.02; *p* = 0.02) were associated with RVD, but the slope of increasing intensity over time was not (OR 1.0; 95% CI: 0.99–1.01; *p* = 0.90). The associations remained after adjustment for age.

The area under the operative curves (AUC) for all univariate and adjusted models are shown in Table 3. The model had an AUC significantly greater statistically when adjusted for the age of the patient. Sensitivity, specificity, predictive values and likelihood ratios at cut-points determined using the Youden index, are given in Table 4. All cut-off points had comparable performance. They had a better ability to rule-in RVD with a positive test (high positive predictive value) than to rule-out RVD with a negative test (low negative predictive value).

**Table 3:** Area under the operative curves (AUC) for the different determinants of the time-intensity curves. Predictive values are given for the RVD assessed on CTA as right-to-left ventricular ratio>1.

	Unadjusted	Age adjusted
Time to inflection point*	0.65 (0.55–0.76)	0.71 (0.61–0.81)
Time to 40HU^†^	0.66 (0.56–0.76)	0.73 (0.63–0.82)
Intensity at 10 s^‡^	0.63 (0.53–0.74)	0.69 (0.59–0.79)
Slope of intensity/time^§^	0.52 (0.42–0.63) ^||^	0.65 (0.54–0.75)**

* Time needed by the contrast media to reach the pulmonary trunk after its injection into the peripheral vein.

^†^ Time needed to reach a determined intensity (40HU) starting from the injection time.

^‡^ The intensity reached at 10 seconds or the highest intensity reached before, when the elapsed time was less than 10 seconds.

^§^ The slope of intensity was calculated by subtracting the intensity measured at the inflection point to the highest intensity measured divided by the time between those two points.

^||^ p<0.05 compared to AUC of intensity at 10 seconds.

** p<0.05 compared to AUC of time to 40HU.

CTA: computed tomography angiography; CI: confidence interval; HU: Hounsfield unit; RVD: right ventricular dysfunction; s: seconds.

**Table 4:** Empirical optimal cut-points of the four parameters of the time-intensity curves.

Parameters of the intensity-time curve	Cut-point	Youden index	Se	Sp	PV+	PV-	LR+	LR-
Time to inflection point*	8	0.29	57	73	70	60	2.1	0.6
Time to 40HU†	11	0.30	54	76	73	58	2.2	0.6
Intensity at 10 s‡	22.9	0.33	53	80	76	59	2.7	0.6
Slope of intensity/time§	35.5	0.15	59	56	61	54	1.3	0.7

* Time needed by the contrast media to reach the pulmonary trunk after its injection into the peripheral vein.

^†^ Time needed to reach a determined intensity (40HU) starting from the injection time.

^‡^ The intensity reached at 10 seconds or the highest intensity reached before, when the elapsed time was less than 10 seconds.

^§^ The slope of intensity was calculated by subtracting the intensity measured at the inflection point to the highest intensity measured divided by the time between those two points.

HU: Hounsfield unit; LR+: positive likelihood ratio; LR-: negative likelihood ratio; PV+: positive predictive value; PV-: negative predictive value; RVD: right ventricular dysfunction; Se: sensitivity; Sp: specificity.

In the sensitivity analysis restricted to RVD assessed by an experienced radiologist, the association between the intensity at 10 seconds and RVD was not statistically significant (S1 and S2 Tables).

## Discussion

The present retrospective proof-of-concept study demonstrates an association between parameters of the time-intensity curve obtained by the CTA bolus tracking system and the diagnosis of right ventricular dysfunction related to pulmonary embolism, as assessed on diagnostic CTA images.

Factors affecting contrast enhancement (and thus time-intensity curves) are related to the contrast delivery protocol (e.g: iodine concentration, flow rate, duration of injection) and to the patient’s own physiology (mainly cardiac output) [31–33]. Significant obstruction of the pulmonary vascular bed by acute PE results in right ventricular dysfunction and pulmonary hypertension [34] with in cases of severe obstruction a low right ventricular blood flow and a cardiovascular collapse [10]. Pulmonary arterial pressures influence the contrast-media bolus propagation speed [35]. Thus, elevated pulmonary arterial pressures and the related low transpulmonary blood flow prolong the time to inflection point, the time to peak, and reduces the intensity obtained 10 seconds after contrast media injection.

Our results are in concordance with previous studies that included acute and chronic pulmonary hypertension, but scarce [35], or no patients with PE [23]. However, the performance of all parameters of the time-intensity curve is low in our study (with a sensitivity to detect RVD around 50%). By contrast, for Davarpanah and colleagues, the time to peak was a substantially better marker to discriminate patient with RVD (AUC of 0.86), than the same time measured in our study (AUC of 0.67). This difference in performance could come from the population studied, the assessment of RVD (echocardiography [23] or right-sided heart catheterisation [35]), the choice of a higher threshold of peak enhancement, and/or a faster injection speed with a higher dose of contrast media (5 ml/s versus 3.5 ml/s in our protocol). Indeed, a lower injection rate may flatten the bolus shape and therefore reduce the precision and accuracy of all CTA bolus markers studied in our study [31]. Still, protocols of contrast media administration in pulmonary embolism may use a flow rate varying from 3 ml/s to 6 ml/s [36].

A patent foramen ovale (PFO), found in 35% of patients with PE [37], might also affect the sensibility of the time-intensity curve to assess RV dysfunction. Indeed, in the setting of a right-to-left intracardiac shunt, one part of the bolus contrast may rapidly reaches the systemic circulation, bypassing by the way pulmonary arteries.

A small retrospective study suggested that characteristic of contrast enhancement over time could correlate with death [24]. However this study used parameters somewhat difficult to extract from the curve in everyday practice, and needed an extra injection of contrast media to build the curves. Indeed, risk of renal injury after iodinate contrast increases with the quantity injected and can complicate CTA already after a small amount [38–40]. Thus, we believe the CTA bolus parameters described in the present study are of interest, since they are easily obtained during any diagnostic CTA without additional image processing (time consuming, especially in an emergency unit) or extra contrast media injection, and may reflect the hemodynamic repercussion of PE that could therefore facilitate PE risk stratification. Our results should promote prospective studies to compare the time-intensity curve against more reliable tools (e.g. echocardiography or invasive measure), its relation with clinical relevant parameters (like admission to ICU, necessity of thrombolysis, and death), or its value in a stepwise approach that could help in choosing patients to go further with risk stratification (CTA right-to-left ventricular ratio, echocardiography or biomarkers).

The present study acknowledges some limitations. Firstly, the reliability of the measurements is limited by the resolution of the tracking system (1 to 2 seconds between two measurements), many artefacts (e.g. respiratory movements, misplacement of the point of interest to measure the time-intensity curve), and the need for the CT technician to start manually and separately the bolus tracking system and the release of contrast media into the veins (lack of an automated system). Secondly, since patient information was limited to those present in the radiology database, we were not able to take into account all potential confounders. However, the time to reach the peak attenuation was not affected by weight (whereas peak enhancement was inversely correlated) in a group of patients with a body weight ranging from 50 to 128 kg [41]. Likewise, gender, age and the size and position of the peripheral catheter could only have minimal impact on the bolus media geometry or time to peak [23, 31]. Thirdly, the low injection rate of the contrast media may have reduced the accuracy of our method to assess right ventricular dysfunction. Fourthly, half of the CTs with a PE diagnosed during the considered period had no contrast time—intensity curve available (or rarely a curve that was unreliable since the 40HU threshold was not reached). During this period, upload of the curve in the computer system by the CT technician was optional. The choice not to upload must be random or linked to the technician's workload. Thus, it is unlikely that this choice is associated with a systematic error that could bias the association between time-intensity curves characteristics and RVD. Finally, we did not use echocardiography to better assess the right heart function, but used a measure of RVD that was available on the same CTA. However the association between right-to-left ventricular ratios, RVD on echocardiography, and prognostic of PE have all been well demonstrated previously. Thus those limitations and a low sensitivity to detect RVD assessed on CTA must be taken into consideration when interpreting our results.

## Conclusion

Parameters of the time-intensity curve obtained by the bolus tracking system are associated with the presence of right heart ventricular dysfunction assessed on CTA images. The time needed by the contrast media to enhance the pulmonary arteries to a predefined threshold (time to peak) is most convenient to measure. However, due to limitations of this pilot investigation (and a low performance of the time-intensity curve), additional studies are needed to evaluate the real potential of the present markers to categorise the severity of PE.

## Supporting Information

S1 Table Characteristics of computed tomography angiography by presence or absence of right ventricular dysfunction (RVD) assessed either by right-to-left ventricular ratio (restricted to the experimented radiologist) or by a combination of right-to-left ventricular ratio > 1 and/or the presence of contrast media in hepatic veins. (DOCX)

S2 Table Area under the operative curves (AUC) for the different determinants of the time-intensity curves. Predictive values are given for the RVD assessed on CTA as right-to-left ventricular ratio>1 or presence of reflux of contrast media into the hepatic veins.
(DOCX)

S1 Checklist STARD Checklist. (DOCX)

## Abbreviations

CTA: computed tomography angiography
HU: Hounsfield units
PE: acute pulmonary embolism
RVD: right ventricular dysfunction

## References

[1] Wicki J, Perneger TV, Junod AF, Bounameaux H, Perrier A. Assessing clinical probability of pulmonary embolism in the emergency ward: a simple score. Archives of internal medicine. 2001;161(1):92–7. Epub 2001/01/09. . 11146703 10.1001/archinte.161.1.92

[2] Konstantinides SV, Torbicki A, Agnelli G, Danchin N, Fitzmaurice D, Galie N, et al 2014 ESC guidelines on the diagnosis and management of acute pulmonary embolism. European heart journal. 2014;35(43):3033–69, 69a–69k. Epub 2014/09/01. 10.1093/eurheartj/ehu283 . 25173341

[3] Le Gal G, Righini M, Roy PM, Sanchez O, Aujesky D, Bounameaux H, et al Prediction of pulmonary embolism in the emergency department: the revised Geneva score. Annals of internal medicine. 2006;144(3):165–71. Epub 2006/02/08. . 16461960 10.7326/0003-4819-144-3-200602070-00004

[4] Jaff MR, McMurtry MS, Archer SL, Cushman M, Goldenberg N, Goldhaber SZ, et al Management of massive and submassive pulmonary embolism, iliofemoral deep vein thrombosis, and chronic thromboembolic pulmonary hypertension: a scientific statement from the American Heart Association. Circulation. 2011;123(16):1788–830. Epub 2011/03/23. 10.1161/CIR.0b013e318214914f . 21422387

[5] Marti C, John G, Konstantinides S, Combescure C, Sanchez O, Lankeit M, et al Systemic thrombolytic therapy for acute pulmonary embolism: a systematic review and meta-analysis. European heart journal. 2015;36(10):605–14. Epub 2014/06/12. 10.1093/eurheartj/ehu218 24917641 PMC4352209

[6] Aujesky D, Roy PM, Verschuren F, Righini M, Osterwalder J, Egloff M, et al Outpatient versus inpatient treatment for patients with acute pulmonary embolism: an international, open-label, randomised, non-inferiority trial. Lancet. 2011;378(9785):41–8. Epub 2011/06/28. 10.1016/S0140-6736(11)60824-6 . 21703676

[7] Goldhaber SZ, Visani L, De Rosa M. Acute pulmonary embolism: clinical outcomes in the International Cooperative Pulmonary Embolism Registry (ICOPER). Lancet. 1999;353(9162):1386–9. Epub 1999/05/05. . 10227218 10.1016/s0140-6736(98)07534-5

[8] Coutance G, Cauderlier E, Ehtisham J, Hamon M, Hamon M. The prognostic value of markers of right ventricular dysfunction in pulmonary embolism: a meta-analysis. Crit Care. 2011;15(2):R103 Epub 2011/03/30. 10.1186/cc10119 21443777 PMC3219376

[9] Sanchez O, Trinquart L, Colombet I, Durieux P, Huisman MV, Chatellier G, et al Prognostic value of right ventricular dysfunction in patients with haemodynamically stable pulmonary embolism: a systematic review. European heart journal. 2008;29(12):1569–77. Epub 2008/05/23. 10.1093/eurheartj/ehn208 . 18495689

[10] John G, Marti C, Poletti PA, Perrier A. Hemodynamic indexes derived from computed tomography angiography to predict pulmonary embolism related mortality. BioMed research international. 2014;2014:363756 Epub 2014/08/26. 10.1155/2014/363756 25147798 PMC4087299

[11] Goldhaber SZ. Echocardiography in the management of pulmonary embolism. Annals of internal medicine. 2002;136(9):691–700. Epub 2002/05/07. . 11992305 10.7326/0003-4819-136-9-200205070-00012

[12] Kang DK, Ramos-Duran L, Schoepf UJ, Armstrong AM, Abro JA, Ravenel JG, et al Reproducibility of CT signs of right ventricular dysfunction in acute pulmonary embolism. AJR American journal of roentgenology. 2010;194(6):1500–6. Epub 2010/05/22. 10.2214/AJR.09.3717 . 20489089

[13] Becattini C, Agnelli G, Germini F, Vedovati MC. Computed tomography to assess risk of death in acute pulmonary embolism: a meta-analysis. The European respiratory journal. 2014;43(6):1678–90. Epub 2014/03/08. 10.1183/09031936.00147813 . 24603813

[14] In E, Aydin AM, Ozdemir C, Sokucu SN, Dagli MN. The efficacy of CT for detection of right ventricular dysfunction in acute pulmonary embolism, and comparison with cardiac biomarkers. Japanese journal of radiology. 2015;33(8):471–8. Epub 2015/06/30. 10.1007/s11604-015-0447-9 . 26118888

[15] Seon HJ, Kim KH, Lee WS, Choi S, Yoon HJ, Ahn Y, et al Usefulness of computed tomographic pulmonary angiography in the risk stratification of acute pulmonary thromboembolism. Comparison with cardiac biomarkers. Circulation journal: official journal of the Japanese Circulation Society. 2011;75(2):428–36. Epub 2010/12/22. . 21173497 10.1253/circj.cj-10-0361

[16] Aribas A, Keskin S, Akilli H, Kayrak M, Erdogan HI, Guler I, et al The use of axial diameters and CT obstruction scores for determining echocardiographic right ventricular dysfunction in patients with acute pulmonary embolism. Japanese journal of radiology. 2014;32(8):451–60. Epub 2014/05/14. 10.1007/s11604-014-0327-8 . 24819998

[17] Meinel FG, Nance JW Jr., Schoepf UJ, Hoffmann VS, Thierfelder KM, Costello P, et al Predictive Value of Computed Tomography in Acute Pulmonary Embolism: Systematic Review and Meta-analysis. The American journal of medicine. 2015;128(7):747–59 e2. Epub 2015/02/15. 10.1016/j.amjmed.2015.01.023 . 25680885

[18] Trujillo-Santos J, den Exter PL, Gomez V, Del Castillo H, Moreno C, van der Hulle T, et al Computed tomography-assessed right ventricular dysfunction and risk stratification of patients with acute non-massive pulmonary embolism: systematic review and meta-analysis. Journal of thrombosis and haemostasis: JTH. 2013;11(10):1823–32. Epub 2013/08/24. 10.1111/jth.12393 . 23964984

[19] Weekes AJ, Thacker G, Troha D, Johnson AK, Chanler-Berat J, Norton HJ, et al Diagnostic Accuracy of Right Ventricular Dysfunction Markers in Normotensive Emergency Department Patients With Acute Pulmonary Embolism. Annals of emergency medicine. 2016 Epub 2016/03/15. 10.1016/j.annemergmed.2016.01.027 . 26973178

[20] George E, Kumamaru KK, Ghosh N, Gonzalez Quesada C, Wake N, Bedayat A, et al Computed tomography and echocardiography in patients with acute pulmonary embolism: part 2: prognostic value. Journal of thoracic imaging. 2014;29(1):W7–12. Epub 2013/10/26. 10.1097/RTI.0000000000000048 . 24157622

[21] Meyer G, Vicaut E, Danays T, Agnelli G, Becattini C, Beyer-Westendorf J, et al Fibrinolysis for patients with intermediate-risk pulmonary embolism. The New England journal of medicine. 2014;370(15):1402–11. Epub 2014/04/11. 10.1056/NEJMoa1302097 . 24716681

[22] Bae KT, Heiken JP, Brink JA. Aortic and hepatic contrast medium enhancement at CT. Part II. Effect of reduced cardiac output in a porcine model. Radiology. 1998;207(3):657–62. Epub 1998/06/04. 10.1148/radiology.207.3.9609887 . 9609887

[23] Davarpanah AH, Hodnett PA, Farrelly CT, Shah SJ, Cuttica M, Ragin AB, et al MDCT bolus tracking data as an adjunct for predicting the diagnosis of pulmonary hypertension and concomitant right-heart failure. AJR American journal of roentgenology. 2011;197(5):1064–72. Epub 2011/10/25. 10.2214/AJR.10.5420 . 22021497

[24] Li C, Lin CT, Kligerman SJ, Hong SN, White CS. Enhancement Characteristics of the Computed Tomography Pulmonary Angiography Test Bolus Curve and Its Use in Predicting Right Ventricular Dysfunction and Mortality in Patients With Acute Pulmonary Embolism. Journal of thoracic imaging. 2015;30(4):274–81. Epub 2015/01/31. 10.1097/RTI.0000000000000141 . 25635705

[25] Reid JH, Murchison JT. Acute right ventricular dilatation: a new helical CT sign of massive pulmonary embolism. Clinical radiology. 1998;53(9):694–8. Epub 1998/10/10. . 9766724 10.1016/s0009-9260(98)80297-3

[26] Ghaye B, Ghuysen A, Willems V, Lambermont B, Gerard P, D'Orio V, et al Severe pulmonary embolism:pulmonary artery clot load scores and cardiovascular parameters as predictors of mortality. Radiology. 2006;239(3):884–91. Epub 2006/04/11. 10.1148/radiol.2392050075 . 16603659

[27] Ghuysen A, Ghaye B, Willems V, Lambermont B, Gerard P, Dondelinger RF, et al Computed tomographic pulmonary angiography and prognostic significance in patients with acute pulmonary embolism. Thorax. 2005;60(11):956–61. Epub 2005/09/01. 10.1136/thx.2005.040873 16131526 PMC1747227

[28] Cuzick J. A Wilcoxon-type test for trend. Statistics in medicine. 1985;4(1):87–90. Epub 1985/01/01. . 3992076 10.1002/sim.4780040112

[29] Gosselin MV, Rubin GD. Altered intravascular contrast material flow dynamics: clues for refining thoracic CT diagnosis. AJR American journal of roentgenology. 1997;169(6):1597–603. Epub 1997/12/11. 10.2214/ajr.169.6.9393173 . 9393173

[30] Aviram G, Rogowski O, Gotler Y, Bendler A, Steinvil A, Goldin Y, et al Real-time risk stratification of patients with acute pulmonary embolism by grading the reflux of contrast into the inferior vena cava on computerized tomographic pulmonary angiography. Journal of thrombosis and haemostasis: JTH. 2008;6(9):1488–93. Epub 2008/07/22. 10.1111/j.1538-7836.2008.03079.x . 18638012

[31] Cademartiri F, van der Lugt A, Luccichenti G, Pavone P, Krestin GP. Parameters affecting bolus geometry in CTA: a review. Journal of computer assisted tomography. 2002;26(4):598–607. Epub 2002/09/10. . 12218827 10.1097/00004728-200207000-00022

[32] Ramos-Duran LR, Kalafut JF, Hanley M, Schoepf UJ. Current contrast media delivery strategies for cardiac and pulmonary multidetector-row computed tomography angiography. Journal of thoracic imaging. 2010;25(4):270–7. Epub 2010/11/03. 10.1097/RTI.0b013e3181efe8b0 . 21042065

[33] Bae KT, Heiken JP, Brink JA. Aortic and hepatic contrast medium enhancement at CT. Part I. Prediction with a computer model. Radiology. 1998;207(3):647–55. Epub 1998/06/04. 10.1148/radiology.207.3.9609886 . 9609886

[34] McIntyre KM, Sasahara AA. The hemodynamic response to pulmonary embolism in patients without prior cardiopulmonary disease. The American journal of cardiology. 1971;28(3):288–94. Epub 1971/09/01. . 5155756 10.1016/0002-9149(71)90116-0

[35] Pienn M, Kovacs G, Tscherner M, Avian A, Johnson TR, Kullnig P, et al Non-invasive determination of pulmonary hypertension with dynamic contrast-enhanced computed tomography: a pilot study. European radiology. 2014;24(3):668–76. Epub 2013/12/07. 10.1007/s00330-013-3067-8 . 24311231

[36] Tillich M, Schoellnast H. Optimized imaging of pulmonary embolism. European radiology. 2005;15 Suppl 5:E66–70. Epub 2008/07/19. . 18637232 10.1007/s10406-005-0167-9

[37] Konstantinides S, Geibel A, Kasper W, Olschewski M, Blumel L, Just H. Patent foramen ovale is an important predictor of adverse outcome in patients with major pulmonary embolism. Circulation. 1998;97(19):1946–51. Epub 1998/06/03. . 9609088 10.1161/01.cir.97.19.1946

[38] Chong E, Shen L, Poh KK, Tan HC. Risk scoring system for prediction of contrast-induced nephropathy in patients with pre-existing renal impairment undergoing percutaneous coronary intervention. Singapore medical journal. 2012;53(3):164–9. Epub 2012/03/22. . 22434288

[39] Azzalini L, Spagnoli V, Ly HQ. Contrast-Induced Nephropathy: From Pathophysiology to Preventive Strategies. The Canadian journal of cardiology. 2015 Epub 2015/08/19. 10.1016/j.cjca.2015.05.013 . 26277092

[40] Manske CL, Sprafka JM, Strony JT, Wang Y. Contrast nephropathy in azotemic diabetic patients undergoing coronary angiography. The American journal of medicine. 1990;89(5):615–20. Epub 1990/11/01. . 2239981 10.1016/0002-9343(90)90180-l

[41] Platt JF, Reige KA, Ellis JH. Aortic enhancement during abdominal CT angiography: correlation with test injections, flow rates, and patient demographics. AJR American journal of roentgenology. 1999;172(1):53–6. Epub 1999/01/15. 10.2214/ajr.172.1.9888738 . 9888738

