# Supplementary material for: Contrast Circulation Time to Assess Right Ventricular Dysfunction in Pulmonary Embolism: A Retrospective Pilot Study
Source: PLoS One. 2016 Aug 23;11(8):e0159674. doi: 10.1371/journal.pone.0159674 (PMC4994948; doi:10.1371/journal.pone.0159674)
Supplement: S1 Table — (DOCX) [file pone.0159674.s001.docx]

| **S1 table:** Characteristics of computed tomography angiography by presence or absence of right ventricular dysfunction (RVD) assessed either by right-to-left ventricular ratio (restricted to the **experimented radiologist**) or by a **combination** of right-to-left ventricular ratio > 1 and/or the presence of contrast media in hepatic veins | | | |
| --- | --- | --- | --- |
|  | **RVD** | **No RVD** | ***P* value** |
| Right-to-left ventricular diameter ratio>1 | N **55** | N **59** |  |
| Mean age (95%CI), y | 73 (57-82) | 66 (50-79) | 0.02 |
| Female (%) | 24 (44%) | 28 (47%) | 0.68 |
| Central PE (%) | 27 (49%) | 17 (29%) | 0.03 |
| Segmental/ sub segmental PE | 28 (51%) | 42 (71%) | 0.03 |
| Median (IQR) time to inflection, s | 8 (7-9) | 7 (6-8) | 0.01 |
| Median (IQR) time to 40UH, s | 11 (8.5-14) | 9.5 (8-11.75) | 0.04 |
| Median (IQR) intensity at 10s, UH | 34.6 (5.9-68.2) | 46.2 (9.6-75.5) | 0.19 |
| Median (IQR) slope of intensity/time, HU/s | 30.5 (20.2-49.8) | 38.8 (22.5-56.9) | 0.18 |
| Combined reflux right-to-left ratio>1 or contrast in hepatic veins | N **65** | N **49** |  |
| Mean age (95%CI), y | 73 (63-81) | 61 (50-76) | 0.01 |
| Female (%) | 9 (50%) | 29 (45%) | 0.70 |
| Central PE (%) | 31 (48%) | 13 (27%) | 0.02 |
| Segmental/ sub segmental PE | 34 (52%) | 36 (73%) | 0.02 |
| Median (IQR) time to inflection^*^, s | 8 (7-9) | 7 (5.5-7.5) | <0.01 |
| Median (IQR) time to 40HU^†^, s | 11 (9-14) | 9.5 (8-10.5) | <0.01 |
| Median (IQR) intensity at 10 seconds^‡^, HU | 19 (3-66.7) | 53.3 (32.8-84.6) | <0.01 |
| Median (IQR) slope of intensity/time^§^, UH/s | 31.4 (20.2-56.2) | 36 (22.5-52.9) | 0.62 |
| * Time needed by the contrast media to reach the pulmonary trunk after its injection into the peripheral vein. † Time needed to reach a determined intensity (40HU) starting from the injection time. ‡ The intensity reached at 10 seconds or the highest intensity reached before, when the elapsed time was less than 10 seconds. § The intensity slope was calculated by subtracting the intensity measured at the inflection point to the highest intensity measured divided by the time between those two points. CTA: computed tomography angiography; CI: confidence interval; HU: Hounsfield unit; IQR: interquartile range 25%-75%; PE: pulmonary embolism; RVD: right ventricular dysfunction; s: seconds; y: years | | | |
