# Supplementary material for: Contrast Circulation Time to Assess Right Ventricular Dysfunction in Pulmonary Embolism: A Retrospective Pilot Study
Source: PLoS One. 2016 Aug 23;11(8):e0159674. doi: 10.1371/journal.pone.0159674 (PMC4994948; doi:10.1371/journal.pone.0159674)
Supplement: S2 Table — Predictive values are given for the RVD assessed on CTA as right-to-left ventricular ratio>1 or presence of reflux of contrast media into the hepatic veins. (DOCX) [file pone.0159674.s002.docx]

| **S2 table:** Area under the operative curves (AUC) for the different determinants of the time-intensity curves. Predictive values are given for the RVD assessed on CTA as right-to-left ventricular ratio>1 or presence of reflux of contrast media into the hepatic veins. | | | | |
| --- | --- | --- | --- | --- |
|  | **Right-to-left ventricular diameter ratio>1** | | **Combined reflux right-to-left ratio>1 or contrast in hepatic veins** | |
|  |  | **Age adjusted** |  | **Age adjusted** |
| Time to inflection point^*^ | 0.64 (0.53-0.74) | 0.67 (0.56-0.77) | 0.68 (0.58-0.78) | 0.72 (0.63-0.82) |
| Time to 40HU^†^ | 0.61 (0.51-0.71) | 0.66 (0.56-0.76) | 0.67 (0.57-0.77) | 0.73 (0.63-0.82) |
| Intensity at 10 s^‡^ | 0.57 (0.46-0.68) ^\|\|^ | 0.64 (0.53-0.74) | 0.65 (0.54-0.76) ^††^ | 0.69 (0.59-0.79) |
| Slope of intensity/time^§^ | 0.57 (0.46-0.68)** | 0.65 (0.54-0.75) | 0.53 (0.42-0.64) ^††^ | 0.65 (0.54-0.75) \|\| |
| * Time needed by the contrast media to reach the pulmonary trunk after its injection into the peripheral vein. † Time needed to reach a determined intensity (40HU) starting from the injection time. ‡ The intensity reached at 10 seconds or the highest intensity reached before, when the elapsed time was less than 10 seconds. § The slope of intensity was calculated by subtracting the intensity measured at the inflection point to the highest intensity measured divided by the time between those two points; \|\| p<0.05 compared to AUC of time to inflection point; ** p<0.05 compared to AUC of time to inflection point and time to 40HU  ^††^ p<0.05 compared to AUC of all other determinants;  CTA: computed tomography angiography; CI: confidence interval; HU: Hounsfield unit; RVD: right ventricular dysfunction; s: seconds. | | | | |
